# [Retracted] Tanshinone IIA improves hypoxic ischemic encephalopathy through TLR‑4‑mediated NF‑κB signal pathway

**DOI:** 10.3892/mmr.2025.13428

**Published:** 2025-01-03

**Authors:** Chengzhi Fang, Lili Xie, Chunmei Liu, Chunhua Fu, Wei Ye, Hong Liu, Binghong Zhang

Mol Med Rep 18: 1899–1908, 2018; DOI: 10.3892/mmr.2018.9227

Following the publication of this paper, it was drawn to the Editor's attention by a concerned reader that the IL-1 protein data shown in the western blotting data in Fig. 5A on p. 1905, the hippocampal images shown in Fig. 6A and certain of the immunohistochemical data shown in Fig. 6B on p. 1906 were strikingly similar to data appearing in different form in other articles written by different authors at different research institutes that had either already been published elsewhere prior to the submission of this paper to *Molecular Medicine Reports*, or were under consideration for publication at around the same time. In view of the fact that certain of the abovementioned data had already apparently been published previously, the Editor of *Molecular Medicine Reports* has decided that this paper should be retracted from the Journal. The authors were asked for an explanation to account for these concerns, but the Editorial Office did not receive a satisfactory reply. The Editor apologizes to the readership for any inconvenience caused.

